# Supplementary material for: A Comprehensive Characterization of Stemness in Cell Lines and Primary Cells of Pancreatic Ductal Adenocarcinoma
Source: Int J Mol Sci. 2022 Sep 14;23(18):10663. doi: 10.3390/ijms231810663 (PMC9503169; doi:10.3390/ijms231810663)
Supplement: Supplementary file 1 [file ijms-23-10663-s001.zip › ijms-1884898-supplementary.pdf]

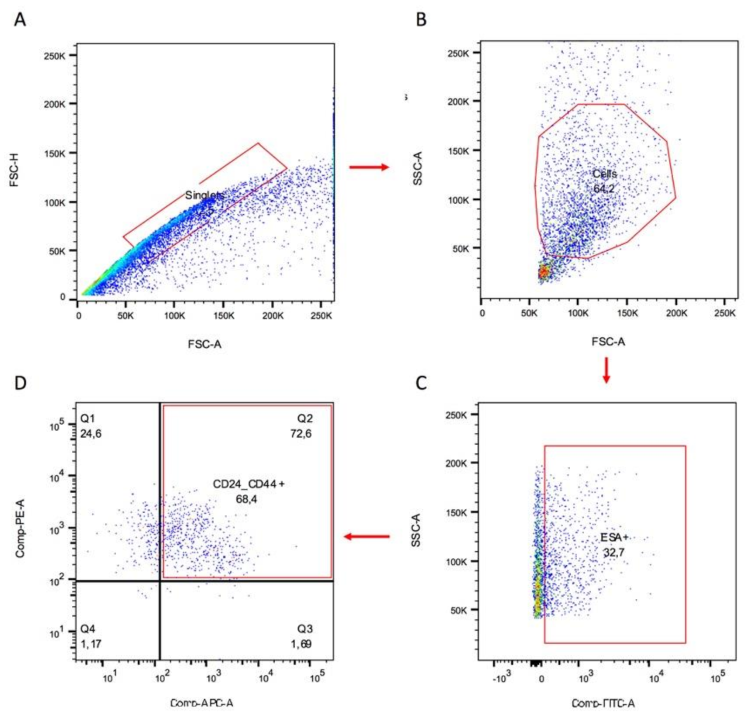

Figure S1. Gating strategy for the stem phenotypes CS44+/CD24+/ESA+. Representative flow cytometry plots read outs of ESA/CD24/CD44 markers: PDAC tumor-derived cells were gated based on the FSC-H/FSC-A (A), then gated based on FSC-A/SSC-A (B), then selected for the marker ESA+ (C) and finally selected for the positivity to CD24 and CD44 markers (D).

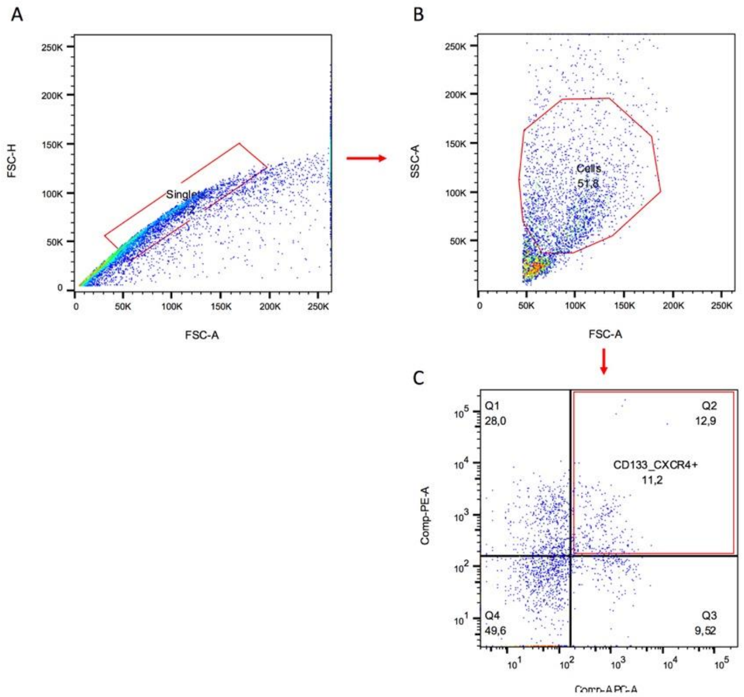

Figure S2. Gating strategy for the stem phenotypes CD133+/CXCR4+. Representative flow cytometry plots read outs of CD133/CXCR4 markers: PDAC tumor-derived cells were gated based on the FSC-H/FSC-A (A), then gated based on FSC-A/SSC-A (B) and then selected for the positivity to CD133 and CXCR4 markers (C).

A

Patient characteristics	
Sex (M/F)	32/23
Age (years; mean±sd)	66.2±10
pT1/T2/T3 [n, (%)]	1 (1.8)/1 (1.8)/53 (96.4)
pN1 [n, (%)]	40 (72.7)
Lymph nodes positive (%)	32.4 (14.22-46.8)
pM1 [n, (%)]	7 (12.7)
R1 [n, (%)]	30 (54.5)
Grading: G1/G2/G3 [n, (%)]	1 (1.8)/32 (58.2)/22 (40)
Tumor size (cm)	2.5 (2-3)
Neo-adjuvant chemotherapy [n, (%)]	3 (5.5)
Adjuvant chemo/radiotherapy [n, (%)]	39 (79.2)
Follow-up (median)	13.1 years
Overall survival [median (95%CI)]	502 (313-691) days
Disease free survival [median (95%CI)]	366 (215-517) days
Local Relapse [n, (%)]	11 (20)
Distant relapse [n, (%)]	26 (47.3)
- Liver	14 (53.8)
- Lung	5 (19.2)
- Lymph nodes	5 (19.2)
- Peritoneal Carcinomatosis	10 (38.5)
- Other sites	3 (11.5)

B

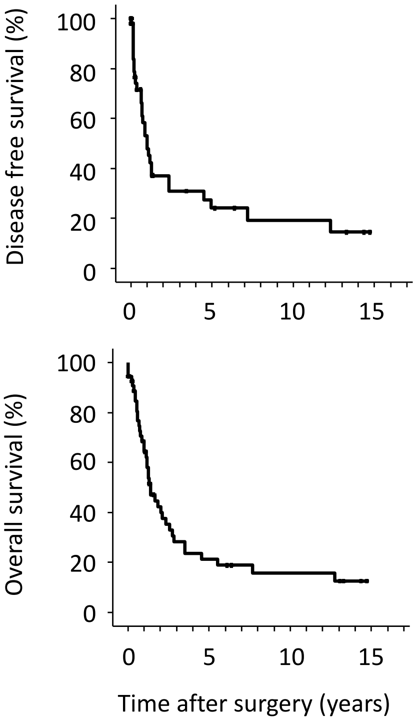

Figure S3. Cohort of 55 patients affected by PDAC. (a) Clinical, pathological and anthropometric characteristics of patients. **(b)** Kaplan-Meier curves representing the overall survival and disease-free survival.

Table S1. Expression of putative stem cell markers ESA, CD24, CD44 and CD133, CXCR4 in 17 lines of PDAC. The numbers are expressed as a percentage (%) of positive cells on the total of the cells analyzed by FACS.

Cell line	Stemness*	ESA+ CD24+ CD44+	ESA+ CD24+ CD44-	ESA+ CD24- CD44-	ESA+ CD24- CD44+	ESA- CD24+ CD44-	ESA- CD24- CD44-	ESA- CD24- CD44+	ESA- CD24+ CD44+	ESA+	CD24+	CD44+	CXCR4+ CD133-	CXCR4+ CD133+	CXCR4- CD133-	CXCR4- CD133+	CXCR4+	CD133+
**MIAPaCa-2**	**LS**	0	0	0.01	0	0	0.73	99.1	0.16	0.01	0.12	99.3	0.09	0	99.9	0	0.09	0
**Hst-766**	**LS**	0.01	0	0	0.12	0	0.74	88.8	10.29	0.13	10.3	99.3	0.56	0.02	99.4	0	0.58	0.0
**Panc-2**	**LS**	0.08	0.04	56.9	42.7	0	0.29	0	0	99.7	0.13	42.7	0.8	0.18	97.2	1.84	0.98	2.02
**Panc-1**	**LS**	0.13	0	16.4	82.7	0	0.73	0	0	99.3	0.13	82.8	0	0.01	99.9	0	0.01	0.01
**HPAF-II**	**LS**	0.31	0	4.6	94.8	0	0.31	0	0	99.7	0.31	95.1	0.48	0.37	99.1	0	0.8	0.37
**PT45**	**LS**	1.3	0	0.02	24.6	0	0.31	73.6	0.19	25.9	1.5	99.7	0.12	0.05	99.8	0	0.2	0.05
**PC**	**LS**	1.5	0	3	94.9	0	0.61	0.03	0	99.4	1.5	96.4	0.29	0.02	99.6	0.05	0.3	0.07
**AsPC-1**	**LS**	2.8	0.06	1.8	94.9	0	0.42	0	0	99.6	2.8	97.7	0.52	0	99.5	0	0.5	0
**BI**	**LS**	3.5	2.2	54.8	39.4	0	0.23	0	0	99.8	5.6	42.8	0.76	0.7	65.2	33.3	1.5	34.0
**PaCa-44**	**MS**	6.9	1.3	32.9	57.4	0.02	1.4	0	0.04	98.5	8.3	64.4	0.22	0.1	94.3	5.4	0.3	5.5
**Capan-1**	**MS**	11.6	2.1	18.7	66.9	0	0.64	0	0	99.4	13.7	78.5	0	0.36	5.07	94.6	0.4	94.9
**T3M4**	**MS**	13.2	0.97	10.5	75.1	0	0.3	0	0	99.7	14.1	88.2	2.23	0.13	97.6	0	2.4	0.13
**SKPC-1**	**MS**	17.4	0	0.8	62.1	0	0.65	11.41	7.6	80.4	25	98.5	0.67	0.21	98.9	0.13	0.9	0.34
**BxPc-3**	**HS**	89.7	2	0.91	4.2	0.38	1.13	0	1.6	96.8	93.7	95.6	1.42	0.1	98.4	0.05	1.5	0.15
**PaCa-3**	**HS**	93.6	6.3	0.06	0.01	0	0	0	0	100	99.9	93.6	0	1.18	12.2	86.6	1.2	87.8
**CFPAC-1**	**HS**	98	0.95	0.16	0.81	0	0.09	0	0.2	99.9	99	98.8	0	1.94	1.53	96.5	1.9	98.5
**A8184**	**HS**	99.4	0.5	0.04	0.07	0	0.02	0	0.02	99.9	99.9	99.4	0.58	0.4	77.0	22	1	22.4

* LS= Low-Stemness cell lines; MS= Medium-Stemness cell lines; HS=High-Stemness cell lines.

Table S2. Expression of CCR and CXCR chemokine receptors by FACS analysis. Data are expressed as a percentage (%) of positive cells.

**Cell line**	**Stemness***	CCR1	CCR2	CCR3	CCR4	CCR5	CCR6	CCR7	CCR8	CCR9	CCR10	CXCR1	CXCR2	CXCR3	CXCR4	CXCR5	CXCR6	CX3CR1	XCR1	CXCL1
**MIAPaCa2**	**LS**	6	2	8	22	39	5	2	1	2	2	3	3	13	2	7	17	5	14	2
**Hst-766**	**LS**	12	1	18	24	47	6	9	2	9	11	24	5	27	3	6	69	0	10	16
**Panc-2**	**LS**	3	3	6	39	14	2	3	3	45	3	18	8	15	14	3	21	2	9	8
**Panc-1**	**LS**	5	4	12	39	22	5	2	1	33	5	16	11	16	3	4	11	3	2	10
**HPAF-II**	**LS**	1	1	7	51	29	1	2	1	5	17	14	1	25	4	1	9	0	16	13
**PT45**	**LS**	0	0	1	4	2	1	1	0	11	1	2	0	3	0	0	1	0	12	0
**PC**	**LS**	0	1	1	27	12	0	0	1	1	1	3	1	24	3	1	2	0	15	1
**AsPC-1**	**LS**	11	3	11	35	44	4	7	2	7	3	11	4	42	1	6	20	1	10	1
**BI**	**LS**	3	2	5	9	7	3	4	1	4	2	6	5	8	8	4	7	2	14	4
**TOT**		3 (0.6-8.3)	1.8 (0.7-3.4)	6.5 (3.2-11.3)	27 (15.5-39.3)	21.5 (9.3-41.4)	2.9 (0.6-5)	2.4 (1.6-5.5)	1.4 (0.9-2)	6.9 (3.3-21.8)	2.7 (1.4-8)	10.7 (3.1-16.7)	3.6 (0.8-6.7)	15.6 (10.6-25.8)	2.6 (1.6-5.7)	3.5 (0.9-6)	11.2 (4.5-20.5)	0.8 (0.1-2.1)	4.7 (2-8.1)	4 (0.9-11.3)
**PaCa-44**	**MS**	4	3	7	54	23	2	3	5	27	2	10	6	14	6	2	12	2	10	4
**Capan-1**	**MS**	1	1	2	12	10	0	0	0	6	7	5	0	8	2	0	2	0	24	0
**T3M4**	**MS**	0	1	20	52	33	0	2	0	49	27	19	0	26	11	3	21	0	13	17
**SKPC-1**	**MS**	4	2	10	20	14	2	2	3	35	12	13	2	15	8	3	7	0	14	2
**TOT**		2.4 (0.24-3.9)	1.7 (0.9-2.9)	8.3 (3.4-17.2)	36 (13.8-53.2)	18.6 (10.7-30.4)	1.2 (0.2-3.3)	1.9 (0.4-2.8)	1.3 (0-4.4)	30.7 (11-45)	9.7 (3.4-23.5)	11.8 (6-17.6)	1.1 (0.1-4.7)	14.8 (9.7-22.9)	6.8 (2.6-10.6)	2.4 (0.8-2.6)	9.4 (3.3-18.6)	0.1 (0-1.7)	3.4 (1.7-7)	3.3 (0.7-13.5)
**BxPc-3**	**HS**	0	0	12	18	15	0	2	1	6	7	12	1	17	2	0	13	5	8	9
**PaCa-3**	**HS**	20	2	2	4	13	2	1	1	2	2	3	2	58	2	1	48	1	14	2
**CFPAC-1**	**HS**	13	9	9	72	55	39	10	10	91	5	59	44	66	44	31	26	16	9	27
**A8184**	**HS**	2	1	4	48	25	3	2	2	23	5	7	3	54	6	5	7	3	21	4
**TOT**		7.2 (0.7-18.4)	1.3 (0.6-7.2)	6 (2.7-11)	33 (7.3-66.3)	20.1 (13.5-47.8)	2.6 (0.9-30)	2 (0.9-7.8)	1.6 (0.7-8.1)	14.5 (3.2-73)	4.6 (2.6-6.2)	9.6 (4.3-47.4)	2.6 (0.9-34)	55.8 (26-64)	4 (2.1-34.4)	3 (0.7-24.6)	19.4 (8.8-42.5)	4 (1.7-13.5)	5.3 (3.2-27.8)	6.6 (2.5-22.4)

* LS= Low-Stemness cell lines; MS= Medium-Stemness cell lines; HS=High-Stemness cell lines.

Table S3. Expression of factors relevant for the biological behavior of cancer cells by FACS analysis. Data are expressed as a percentage (%) of positive cells.

**Cell line**	**Stemness***	CD220	CD221	Tie2	CD117	CD15	CD49a	CD49e	CD318	E-cadherin,	CA19-9	CD142	CD200	CD73	CD166	CD105	CD31	CD34	Stro 1
**MIAPaCa2**	**LS**	54	100	1	69	1	1	95	38	23	10	0	0	95	-1	93	6	2	6
**Hst-766**	**LS**	2	84	30	9	2	9	62	94	5	29	83	0	80	78	77	12	9	7
**Panc-2**	**LS**	1	99	7	18	10	37	90	100	38	62	94	0	95	95	43	6	6	6
**Panc-1**	**LS**	2	29	3	60	4	13	98	93	9	93	1	0	97	73	97	8	2	10
**HPAF-II**	**LS**	2	61	2	6	7	12	16	100	64	45	99	3	40	67	2	2	1	2
**PT45**	**LS**	22	99	5	8	0	84	91	59	4	46	96	1	91	91	89	4	4	4
**PC**	**LS**	7	55	7	6	2	0	20	81	4	76	2	0	91	19	53	8	5	6
**AsPC-1**	**LS**	27	100	9	4	1	2	21	96	6	97	46	0	91	43	11	3	2	2
**BI**	**LS**	1	13	3	3	14	4	1	91	2	93	14	0	76	84	10	6	0	3
**Tot**		2.2 (1.2-24.4)	84 (42-99)	5.3 (3.1-25.1)	8 (5-39)	1.8 (1.-8.1)	8.5 (1.5-25.1)	61.7 (18.4-92.8)	93 (70-98)	6.4 (4.3-30.6)	62 (37-93)	46 (1.3-95)	0 (0-0.5)	91 (78-95)	73 (31-88)	53 (10-91)	5.5 (3.2-7.9)	2.1 (1.2-5.4)	5.7 (2.2-6.5)
**PaCa-44**	**MS**	4	78	4	25	9	34	90	99	9	90	84	0	90	89	84	5	2	7
**Capan-1**	**MS**	0	39	2	6	41	66	64	93	67	48	12	0	85	92	6	2	2	1
**T3M4**	**MS**	0	97	5	1	0	17	85	100	11	0	97	0	85	88	49	5	0	1
**SKPC-1**	**MS**	1	99	2	16	0	65	75	100	25	95	91	1	96	95	84	4	2	11
**Tot**		1 (0.1-3.6)	88 (49-99)	3.1 (1.8-5)	10 (2-23)	4.5 (0.1-33)	49.1 (21-65.3)	80 (66.4-88.5)	100 (95-100)	18 (9.6-56.2)	69 (12-93)	88 (30-96)	0 (0-0.4)	87 (85-95)	90 (88-95)	66 (17-84)	4.4 (2.4-4.8)	1.7 (0.4-2.1)	3.8 (0.8-9.9)
**BxPc-3**	**HS**	1	82	4	3	0	11	73	99	44	71	96	0	88	23	2	1	1	1
**PaCa-3**	**HS**	43	100	5	11	5	96	96	96	64	77	0	0	95	96	7	5	3	4
**CFPAC-1**	**HS**	10	98	45	18	3	86	89	100	28	31	49	0	93	95	46	10	2	16
**A8184**	**HS**	38	49	72	19	0	74	74	100	15	94	0	0	94	91	31	2	0	2
**TOT**		23.7 (2.9-41.4)	90 (58-99)	26 (4.2-65)	14 (5-19)	1.6 (0.21-4.6)	80.1 (26.5-93.6)	82 (73-94)	100 (97-100)	36 (18.5-59)	74 (41-90)	25 (0.1-84)	0 (0-0.2)	93 (89-95)	93 (40-96)	19 (3.2-42)	3.2 (0.9-9)	1 (0.1-2.9)	3 (1-13)

* LS= Low-Stemness cell lines; MS= Medium-Stemness cell lines; HS=High-Stemness cell lines.

Table S4. Human PDAC cell lines.

Name	Origin	Supplier	Stabilized by	Patient		
				**Sex**	**Age (yrs)**	**Grade**
Panc-1	Primary tumor	ATCC	M. Lieber (US)	M	56	3
PT45	Primary tumor	N. Lemoine	H. Yamada (Japan)	-	-	3
MIAPaCa-2	Primary tumor	ATCC	A. Yunis	M	65	3
SKPC-1	Primary tumor	A. Scarpa	M.R. Vila (Spain)	M	-	-
BI	Primary tumor	Andren-Sandberg	Andren-Sandberg	-	-	-
PC	Primary tumor	A. Scarpa	Andren-Sandberg	-	-	-
Panc-2	Primary tumor	A. Scarpa	M.v Bulow	F	70	3
PaCa-3	Primary tumor	A. Scarpa	M.v Bulow	M	-	3
PaCa-44	Primary tumor	M.v Bulow	M.v Bulow	F	44	2
BxPc-3	Primary tumor	ATCC	M.H. Tan	F	61	2
Capan-1	Liver metastasis	A. Scarpa	Kyriazis AP	M	40	1
CFPAC-1	Liver metastasis	N. Lemoine	R.A. Schoumacher	M	26	1
Hst-766	Lymph node metastasis	ATCC	Smith HS	M	64	2
T3M4	Lymph node metastasis	N. Lemoine	T. Okabe	M	64	2
A8184	Ascites	ATCC	-	F	75	2
HPAF-II	Ascites	ATCC	Metzgar R.S.	M	44	2
AsPC-1	Ascites	ATCC	Chen and M.H. Tan	F	62	2

Table S5. Antibodies used for FACS analysis.

	Marker	Clone	Species	Isotype	Conjugate	Vendor
**CA19-9**	sialyl-Lewis^{A}	241	Monoclonal, mouse	IgG1	none	GeneTex, Inc., Irvine CA, USA
**CD133/1**	prominin-1	AC133	Monoclonal, mouse	IgG1	PE	Miltenyi Biotec, Germany
**ESA**	epithelial cell adhesion molecule (EpCAM)	9C4	Monoclonal, mouse	IgG	FITC	Abcam, Cambridge, USA
**CD24**	Small Cell Lung Carcinoma Cluster 4 Antigen	ML5	Monoclonal, mouse	IgG2a	PE	BD Bioscience, CA
**CD44**	P-glycoprotein 1	G44-26	Monoclonal, mouse	IgG2b	APC	BD Bioscience, CA
**CCR1**	C-C Motif Chemokine Receptor 1	53504	Monoclonal, mouse	IgG2b	PE	R&D Systems,Minneapolis,MN
**CCR2**	C-C Motif Chemokine Receptor 2	48607	Monoclonal, mouse	IgG2b	PE	R&D Systems,Minneapolis,MN
**CCR3**	C-C Motif Chemokine Receptor 3	61828	Monoclonal, mouse	IgG2a	PE	R&D Systems,Minneapolis,MN
**CCR4**	C-C Motif Chemokine Receptor 4	205410	Monoclonal, mouse	IgG2b	PE	R&D Systems,Minneapolis,MN
**CCR5**	C-C Motif Chemokine Receptor 5	CTC5	Monoclonal, mouse	IgG1	PE	R&D Systems,Minneapolis,MN
**CCR6**	C-C Motif Chemokine Receptor 6	53103	Monoclonal, mouse	IgG2b	PE	R&D Systems,Minneapolis,MN
**CCR7**	C-C Motif Chemokine Receptor 7	150503	Monoclonal, mouse	IgG2a	PE	R&D Systems,Minneapolis,MN
**CCR8**	C-C Motif Chemokine Receptor 8	191704	Monoclonal, mouse	IgG2b	PE	R&D Systems,Minneapolis,MN
**CCR9**	C-C Motif Chemokine Receptor 9	112509	Monoclonal, mouse	IgG2a	PE	R&D Systems,Minneapolis,MN
**CCR10**	C-C Motif Chemokine Receptor 10	314305	Monoclonal, mouse	IgG2a	APC	R&D Systems,Minneapolis,MN
**CXCR1**	C-X-C Motif Chemokine Receptor 1	42705	Monoclonal, mouse	IgG2a	PE	R&D Systems,Minneapolis,MN
**CXCR2**	C-X-C Motif Chemokine Receptor 2	48311	Monoclonal, mouse	IgG2a	PE	R&D Systems,Minneapolis,MN
**CXCR3**	C-X-C Motif Chemokine Receptor 3	49801	Monoclonal, mouse	IgG1	PE	R&D Systems,Minneapolis,MN
**CXCR4**	C-X-C Motif Chemokine Receptor 4	12G5	Monoclonal, mouse	IgG2a	PE	R&D Systems,Minneapolis,MN
**CXCR5**	C-X-C Motif Chemokine Receptor 5	51505	Monoclonal, mouse	IgG2b	PE	R&D Systems,Minneapolis,MN
**CXCR6**	C-X-C Motif Chemokine Receptor 2	56811	Monoclonal, mouse	IgG2b	PE	R&D Systems,Minneapolis,MN
**CX3CR1**	C-X3-C Motif Chemokine Receptor 1	2A9-1	Monoclonal, mouse	IgG2b	PE	MBL, Japan
**XCR1**	X-C Motif Chemokine Receptor 1	AAIL01	Polyclonal, goat	IgG	PE	R&D Systems,Minneapolis,MN
**CD220**	Insulin receptor	3B6/IR	Monoclonal, mouse	IgG1,k	PE	BD Bioscience, CA
**CD221**	Insulin-like growth factor	1H7	Monoclonal, mouse	IgG1,k	PE	BD Bioscience, CA
**Tie2**	Epidermal growth factor homology domain 2	83715	Monoclonal, mouse	IgG	APC	R&D Systems,Minneapolis,MN
**CD15**	Lewis x	HI98	Monoclonal, mouse	IgM,k	FITC	BD Bioscience, CA
**CD318**	CUB domain-containing protein 1	CUB1	Monoclonal, mouse	IgG2b	APC	Biolegend, CA
**CD142**	Tissue Factor	CLB/TF-5	Monoclonal, mouse	IgG1	FITC	Novus Biologicals, USA
**CD200**	OX2 membrane glycoprotein	MRC OX-104	Monoclonal, mouse	IgG1,k	PE	BD Bioscience, CA
**CD166**	Activated Leukocyte Cell Adhesion Molecule (ALCAM)	105902	Monoclonal, mouse	IgG	PE	R&D Systems,Minneapolis,MN
**CD117**	Stem Cell Factor Receptor, C-Kit	YB5.B8	Monoclonal, mouse	IgG1,k	APC	BD Bioscience, CA
**CD105**	Endoglin	166707	Monoclonal, mouse	IgG	FITC	R&D Systems,Minneapolis,MN
**CD49a**	Integrin alpha-1	SR84	Monoclonal, mouse	IgG1,k	PE	BD Bioscience, CA
**CD49e**	Integrin alpha-5	IIA1	Monoclonal, mouse	IgG1,k	PE	BD Bioscience, CA
**CD73**	Ecto-5′-nucleotidase	AD2	Monoclonal, mouse	IgG1,k	PE	BD Bioscience, CA
**CD31**	Platelet endothelial cell adhesion molecule (PECAM-1)	WM59	Monoclonal, mouse	IgG1,k	FITC	BD Bioscience, CA
**Stro-1**	Platelet endothelial cell adhesion molecule (PECAM-1)	STRO-1	Monoclonal, mouse	IgM	none	BD Bioscience, CA
**CD324**	E-cadherin	180224	Monoclonal, mouse	IgG2b	APC	R&D Systems,Minneapolis,MN

Table S6. Gene expression assays (Applied Biosystems) used for expression analysis.

**Gene name**	**Protein**	**Assay number**
CHGA	Chromogranin A	Hs00154441_m1
CHGB	Chromogranin B	Hs00174956_m1
GAD2	Glutamate decarboxylase 2	Hs00609529_m1
GCG	Glucagon	Hs00174967_m1
INS	Insulin	Hs00356618_m1
ISL1	Insulin gene enhancer protein ISL-1	Hs00158126_m1
ONECUT1	One cut homeobox 1	Hs00413554_m1
KRT19	Keratin, type I cytoskeletal 19	Hs00761767_s1
NEUROD1	Neurogenic differentiation factor 1	Hs00139598_m1
NGN3	Neurogenin-3	Hs00360700_g1
NKX2.2	NK2 Homeobox 2	Hs00159616_m1
NKX6.1	Homeobox protein Nkx-6.1	Hs00232355_m1
PAX4	Paired box protein Pax-4	Hs00927346_g1
PAX6	Paired box protein Pax-6	Hs00242217_m1
PDX1	Pancreas/duodenum homeobox protein 1	Hs00426216_m1
PTF1-alpha	Pancreas transcription factor 1 subunit alpha	Hs00603586_g1
SYP	Synaptophysin	Hs00300531_m1
GAPDH	Glyceraldehyde-3-phosphate dehydrogenase	Hs99999905_m1
HNF1-beta	Hepatocyte nuclear factor 1 beta	Hs00172123_m1
SOX17	SRY-box transcription factor 17	Hs00751752_s1
NES	Nestin	Hs00707120_s1
SNAI1	Snail family transcriptional repressor 1	Hs00195591_m1
VIM	Vimentin	Hs00185584_m1
